# Ring finger protein 128 promotes, rather than inhibits, colorectal cancer progression by regulating the Hippo signaling pathway

**DOI:** 10.3389/fonc.2022.1031160

**Published:** 2022-12-29

**Authors:** Shili Ning, Yuzhuo Chen, Guangzhi Wang, Yongtai Liu, Yingchi Yang, Zhongtao Zhang

**Affiliations:** ^1^ Department of General Surgery, Beijing Friendship Hospital, Capital Medical University, Beijing, China; ^2^ Department of General Surgery, Second Hospital of Dalian Medical University, Dalian, China; ^3^ Beijing Key Laboratory of Cancer Invasion and Metastasis Research & National Clinical Research Center for Digestive Diseases, Beijing, China

**Keywords:** RNF128, Hippo signaling pathway, colorectal cancer, cell proliferation, cell invasion and migration

## Abstract

**Background:**

Colorectal cancer is a common malignancy of the gastrointestinal tract, and its incidence and mortality rates have increased in recent years. RNF128 is an E3 ubiquitin ligase that plays an important role as a suppressor gene or oncogene in various cancers, but its mechanism in colorectal cancer is not yet clear. The aim of this study was to investigate the role and mechanism of RNF128 in colorectal cancer.

**Methods:**

The expression of RNF128 in colorectal cancer tissues was assessed by immunohistochemistry and western blotting. The proliferation ability of colorectal cancer cells was measured by colony formation assay and CCK-8 assay, the migration and invasion ability of colorectal cancer cells was measured by wound healing assay and transwell assay, and the protein expression levels of the Hippo signaling pathway and its target gene were examined by western blotting. Immunoprecipitation was used to assess the interaction of RNF128 with MST. *In vivo*, a xenograft tumor model was used to detect the effect of RNF128 on tumor growth.

**Results:**

At the tissue level, the expression level of RNF128 was significantly higher in colorectal cancer tissues than in adjacent normal tissues. In LoVo cells and HCT116 cells, the proliferation, migration and invasion abilities were significantly reduced with RNF128 knockdown. At the protein level, knockdown of RNF128 resulted in significant activation of the Hippo signaling pathway. *In vivo* experiments, the volume and weight of xenograft tumors in nude mice were significantly decreased compared with those in the normal control group with RNF128 knockdown.

**Conclusion:**

RNF128 promotes the malignant behaviors of colorectal cancer cells by inhibiting the Hippo signaling pathway, which may provide a new target for colorectal cancer prevention and treatment.

## Introduction

Colorectal cancer (CRC) is one of the most common cancers worldwide; it ranks third in incidence and second in mortality (1). According to predictions, there will be 2.2 million new cases and 1.1 million mortalities by 2030 (2). According to previous reports, multiple acquired somatic genomic and epigenetic alterations contribute to the initiation and development of CRC (3). To date, the detailed mechanisms of CRC occurrence and development are still unclear.

Ring finger protein 128 (RNF128) is a member of the ubiquitin-protein enzyme (E3) family and was initially also known as a gene related to anergy in lymphocytes due to its ability to suppress T-cell activation and regulate autoimmunity (4–6). Recent studies found that RNF128 can interact with PPARγ to regulate adipogenesis and diet-induced obesity (7), and it can also promote hepatic steatosis by inhibiting sirt1 (8). Recently, aberrant expression of RNF128 has been identified in several tumors, for example, RNF128 regulates tumorigenesis by interacting with p53 and regulating its degradation (9). In esophageal squamous cell carcinoma and hepatocellular carcinoma, RNF128 promotes malignant tumor behavior through activation of the EGFR/ERK signaling pathway (10, 11). However, in melanoma, upper gastrointestinal tract and bladder urothelial carcinoma, RNF128 is downregulated and associated with a poor prognosis (12, 13). Although a previous report demonstrated that RNF128 is downregulated in colorectal cancer tissues and inhibits CRC progression (14), the role and mechanism of RNF128 in colorectal cancer are still controversial. There is strong evidence, including data from the Gene Expression Profiling Interactive Analysis (GEPIA) and the Cancer Genome Atlas (TCGA) databases, showing that RNF128 expression is elevated rather than decreased in colorectal cancer tissues. Furthermore, the results of our preliminary analysis are consistent with the GEPIA and TCGA findings. Thus, we intend to perform further experiments to investigate the role of RNF128 in colorectal cancer.

The Hippo pathway is highly conserved and was initially discovered in *Drosophila* (15, 16). In mammals, the Hippo pathway consists of mammalian STE20-like kinase (MST1/2), large tumor suppressor kinase (LATS1/2), and their adaptor proteins MOB1 and Salvador (SAV1) (17, 18). Yes-associated protein (YAP) and transcriptional coactivator with PDZ-binding motif (TAZ) are two key downstream effectors in the Hippo kinase cascade pathway (19–21). The Hippo kinase cascade inhibits YAP/TAZ entry into the nucleus and binding to the TEA domain family member (TEAD) family by MST/LATS kinase phosphorylation, thereby inhibiting nuclear transcription (22). Thus, the Hippo signaling pathway plays an important role as a suppressor pathway in many cancer processes, such as breast, liver and colorectal cancer (23–26). A previous study on breast cancer showed that RNF family proteins control the malignant biological behavior of breast cancer cells by regulating the Hippo signaling pathway (27). In this study, we used StarBase (https://starbase.sysu.edu.cn/starbase2/), which predicted a strong correlation between RNF128 and the Hippo signaling pathway, and verified this correlation in a colorectal cancer model. Given the important suppressor role of the Hippo signaling pathway in colorectal cancer, we hypothesize that RNF128 may be an important regulator of the Hippo signaling pathway and has an important role in CRC.

In this study, we found that RNF128 was highly expressed in CRC and promoted the proliferation, migration and invasion of colorectal cancer cells. Furthermore, we found that RNF128 promoted CRC progression by inhibiting the Hippo signaling pathway. In conclusion, our findings provide a novel therapeutic target for CRC.

## Materials and methods

### Tissue collection

Colorectal cancer tissues and adjacent normal tissues were obtained from the six patients who underwent surgery for CRC at the Second Hospital of Dalian Medical University. Clinical and pathological information of six CRC patients was in **Table 1**. All patients signed informed consent forms and agreed to the use of their tissues for clinical studies. The study was approved by the Ethical Committee of the Second Hospital of Dalian Medical University.

**Table 1:** Clinical and pathological information of six CRC patients.

Number	Age (years)	Sex	Clinical information	Pathological information
1	72	Female	Colon cancer (pT3N1M0)	Moderately differentiated adenocarcinoma
2	62	Female	Colon cancer (pT3N1M0)	Moderately differentiated adenocarcinoma
3	53	Female	Rectal cancer (pT4N1M0)	Moderately to poorly differentiated adenocarcinoma
4	60	Male	Colon cancer (pT3N1M0)	Moderately differentiated adenocarcinoma
5	58	Male	Colon cancer (pT3N0M0)	Moderately differentiated adenocarcinoma
6	64	Male	Colon cancer (pT3N0M0)	Moderately differentiated adenocarcinoma

### Plasmids and lentivirus infection

RNF128-siRNA and RNF128-shRNA were used to knock down RNF128 expression and synthesized by GenePharma company (Suzhou, China). The sequences of siRNF128 were as follows: siRNF128-1: 5’-GGG UGC AGU AGA CAU UGU UTT-3’; siRNF128-2: 5’-GCA UCA UCU GGA UAU GCU UTT-3’; and si-NC: 5’-UUC UCC GAA CGU GUC ACG UTT-3’. Lipofectamine 3000 (L3000015, Thermo Fisher) was used for transfection in HCT116 and LoVo cell lines. The sequence of shRNF128 was 5’-GGG TGC AGT AGA CAT TGT T-3’; the sequence of shNC was 5’-TTC TCC GAA CGT GTC ACG T-3’.

### Cell lines and cell culture

LoVo and HCT116 cells were purchased from the Type Culture Collection of the Chinese Academy of Science (Shanghai, China). LoVo cells were cultured in RPMI 1640 cell culture (Gibco) containing 10% fetal bovine serum, and HCT116 cells were cultured in McCoy’s 5A (Gibco) containing 10% fetal bovine serum and cultured in a 5% CO_2_ incubator at 37°C. The proteasome inhibitor MG132 (MedChemExpress, HY-13259) was used to treat HCT116 cells for 6 h, the YAP inhibitor verteporfin (TargetMol, T3112) was used to treat HCT116 cells for 16 h, and the YAP agonist PY-60 (TargetMol, T9566) was used to treat HCT116 cells for 24 h.

### Cell Counting Kit-8 assay

HCT116 and LoVo cells were transfected with siRNF128 and siControl. After 24 h of transfection, the cell number was counted, and the cells were plated into 96-well dishes at a density of 3×10^3^ cells per well. Ten microliters of CCK-8 solution was added to each well (Meilunbio, MA0218-2) and incubated for 2 h at 37°C. The OD value of each well was read at 450 nm.

### Colony formation assay

LoVo and HCT116 cells were transfected with siRNF128 and siControl for 24 h, counted and seeded at 1×10^3^ cells per well in 6-well plates, and cultured in 5% CO_2_ at 37°C for one week. Cells were fixed with 10% methanol and stained with 0.1% crystal violet.

### Wound healing assay

HCT116 and LoVo cells were transfected with siRNF128 and siControl. HCT116 and LoVo cells were plated in 6-well plates at a seeding density of 5×10^5^ and cultured until 90% confluence. A sterile 10-µl pipette tip was used to make scratch wounds, and cell debris was washed off using PBS buffer. RPMI 1640 and McCoy’s 5A medium with 2% FBS were added to the cells, and scratch lines were photographed and measured under a microscope at 0 h and 48 h.

### Migration and invasion assays

A total of 1×10^5^ cells were added to a Transwell chamber (Corning, Cat3422) with or without Matrigel. The lower chamber contained 600 µl RPMI-1640 and McCoy’s 5A medium with 20% FBS. After 24 h, the invaded cells on the lower surface of the membrane were fixed with 100% methanol and stained with 0.1% crystal violet, and six fields were randomly selected in each group for analysis.

### H&E staining

The colorectal cancer tissues were fixed with paraformaldehyde for 24 h, dehydrated, embedded in paraffin, and sectioned at 4 μm. Paraffin sections were dewaxed and hydrated and stained with hematoxylin/eosin for analysis.

### Immunohistochemistry

The colorectal cancer tissues and tumor tissues were collected, and paraffin-embedded sections were prepared as described above. Antigen retrieval was performed using citrate buffer (pH 6.0), and endogenous peroxidase activity was blocked with serum for 30 min at room temperature. Primary antibodies were added and incubated overnight, secondary antibodies were incubated for 50 min at room temperature, DBA was used for color development (positive cells showed brown color), and the nuclei were counterstained with hematoxylin. The sections were acquired and analyzed.

### Western blot and antibodies

Lysis buffer was added to cells or tissues. The antibodies used were as follows: RNF128 (Abcam 72533, 1:1000), MST (ZEN-BIOSCIENCE 382248, 1:1000; Proteintech 22245-1-AP, 1:1000), p-LATS (Affinity AF8163, 1:1000), YAP (Affinity DF3182, 1:1000), p-YAP (Affinity AF3328, 1:1000), TAZ (Proteintech 23306-1-AP, 1:1000), β-actin (Bimake A5092, 1:2000), IgG (ABclonal AC005), Bcl-2 (Proteintech 60178-1-Ig, 1:1000), HIF-1α (Ploneer in Proteomics PTM-5851, 1:1000), IκBα (Proteintech 10268-1-AP, 1:1000), p-IκBα (Santa Cruz Biotechnology sc-8404, 1:500) and Ub (Proteintech 10201-2-AP, 1:1000). The primary antibody was incubated with the blots for 24 h at 4°C, and the secondary antibody was incubated for 2 h at 37°C.

### Immunoprecipitation

RNF128, MST and IgG antibodies were added to the protein A/G beads (Bimake B23201) and mixed at room temperature for 3 h, and then the protein supernatant was added and mixed at room temperature for 3 h. Western blotting was used for analysis of the bound proteins.

### Xenograft tumor formation

Athymic nude mice (4-6 weeks old, male) were purchased from GemPharmatech company. All mice used for experiments were approved by the Institutional Animal Care and Use Committee of The Second Hospital of Dalian Medical University. The mice were randomly divided into two groups (5 mice per group) and injected with LoVo cells that were transfected with shNC and shRNF128. Tumors were weighed and measured, and volume was calculated as follows: V=ab^2^/2. After 28 days, tumors were removed and used for IHC.

### Statistical analysis

All data are the average of three independent experiments ± standard deviation (SD). Statistical analyses were performed using GraphPad Prism (version 8; GraphPad Software). Differences between two groups were analyzed by Student’s t test. *P* values <0.05 were considered statistically significant (*P<0.05, **P<0.001, ***P<0.0001).

## Results

### RNF128 is highly expressed in colorectal cancer tissues

**Figure 1:**
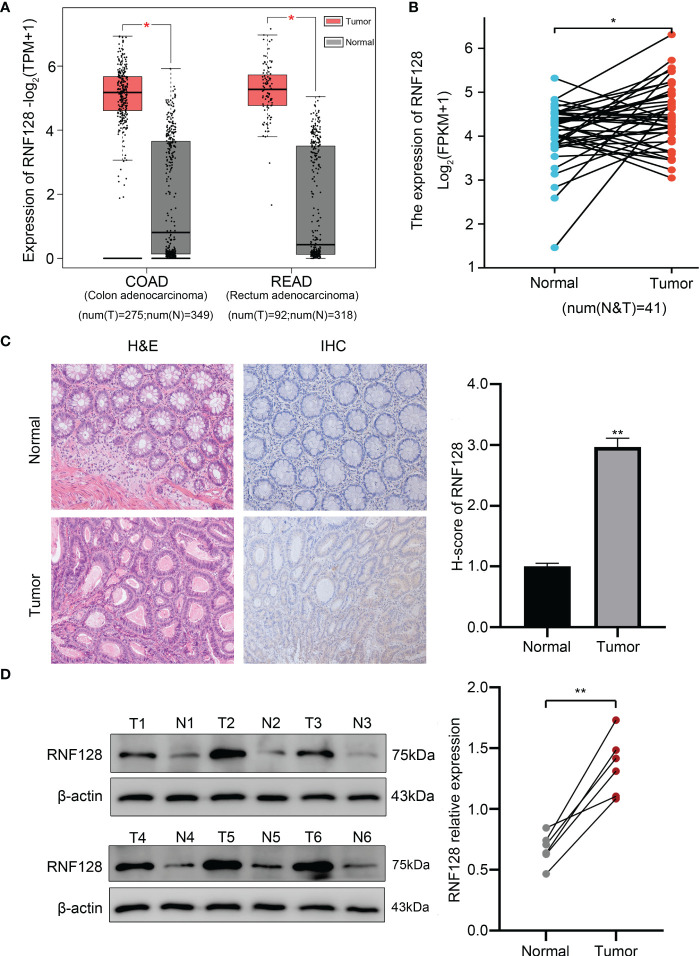
RNF128 is upregulated in colorectal cancer tissues. **(A, B)** The mRNA expression of RNF128 was evaluated in CRC datasets from the GEPIA and TCGA databases. **(C)** HE and IHC analysis of RNF128 expression in normal tissues and CRC tissues. **(D)** The protein levels of RNF128 in CRC tissues and normal tissues (n=6) were analyzed by western blot assays. *P<0.05, **P<0.001.

To explore the differences in the expression of RNF128 in CRC and normal tissues, we assessed the mRNA expression levels by the GEPIA database, which showed that RNF128 was expressed at higher levels in CRC tissues than in adjacent normal tissues (**Figure 1A**). Furthermore, we analyzed the level of RNF128 mRNA in CRC paired samples by TCGA database, and the results showed that the level of RNF128 mRNA was higher in colorectal cancer tissues (**Figure 1B**). To further verify the expression of RNF128 in CRC tissues, IHC and western blot assays were carried out, and the results showed that the protein expression of RNF128 was significantly elevated (**Figures 1C, D**). All the above results demonstrated that RNF128 expression is increased, rather than decreased, in colorectal cancer tissues.

### Knockdown of RNF128 inhibits CRC cell proliferation

**Figure 2:**
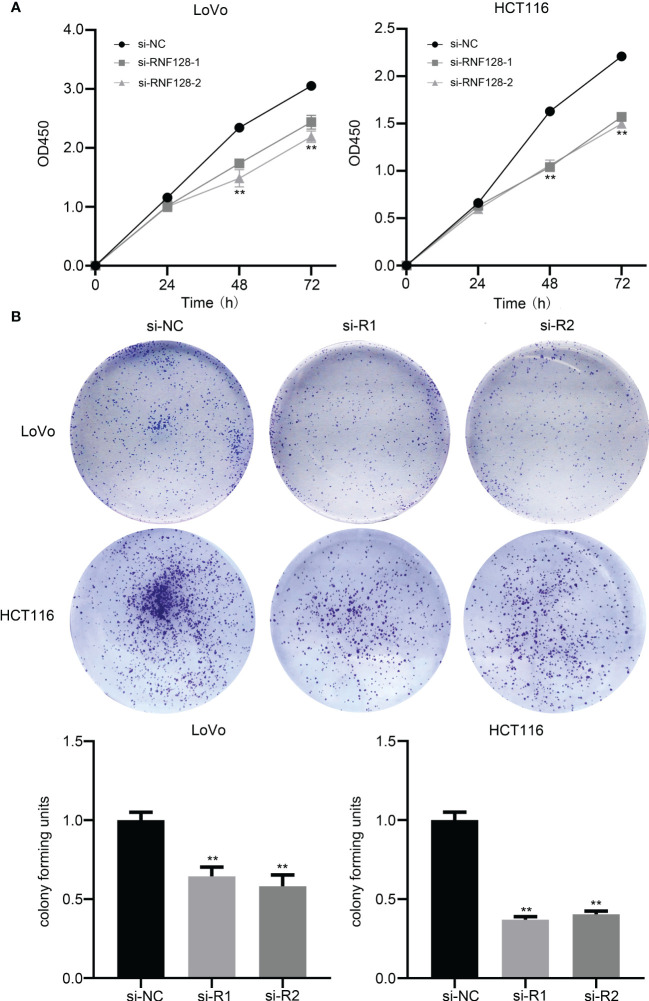
RNF128 promotes cell proliferation in colorectal cancer. **(A)** Cell proliferation was analyzed by CCK-8 assay, and cell viability was detected at 24, 48, and 72 h. LoVo and HCT116 cells were transfected with si-NC and si-RNF128. **(B)** Cell colony formation assays were used to observe the tumor formation ability of LoVo and HCT116 cells transfected with si-NC and si-RNF128. **P<0.001.

To evaluate the effect of RNF128 expression levels on colorectal cancer cell growth, we used small interfering RNA (siRNA) to knockdown RNF128 in LoVo and HCT116 cell lines. Proliferative capacity was assessed by CCK-8 and colony formation assays. The results showed that cell proliferation was suppressed and the number of CFUs was decreased with RNF128 knockdown (**Figures 2A, B**). Thus, RNF128 might be a proliferation-promoting factor in CRC cells.

### Knockdown of RNF128 inhibits CRC cell migration and invasion

**Figure 3:**
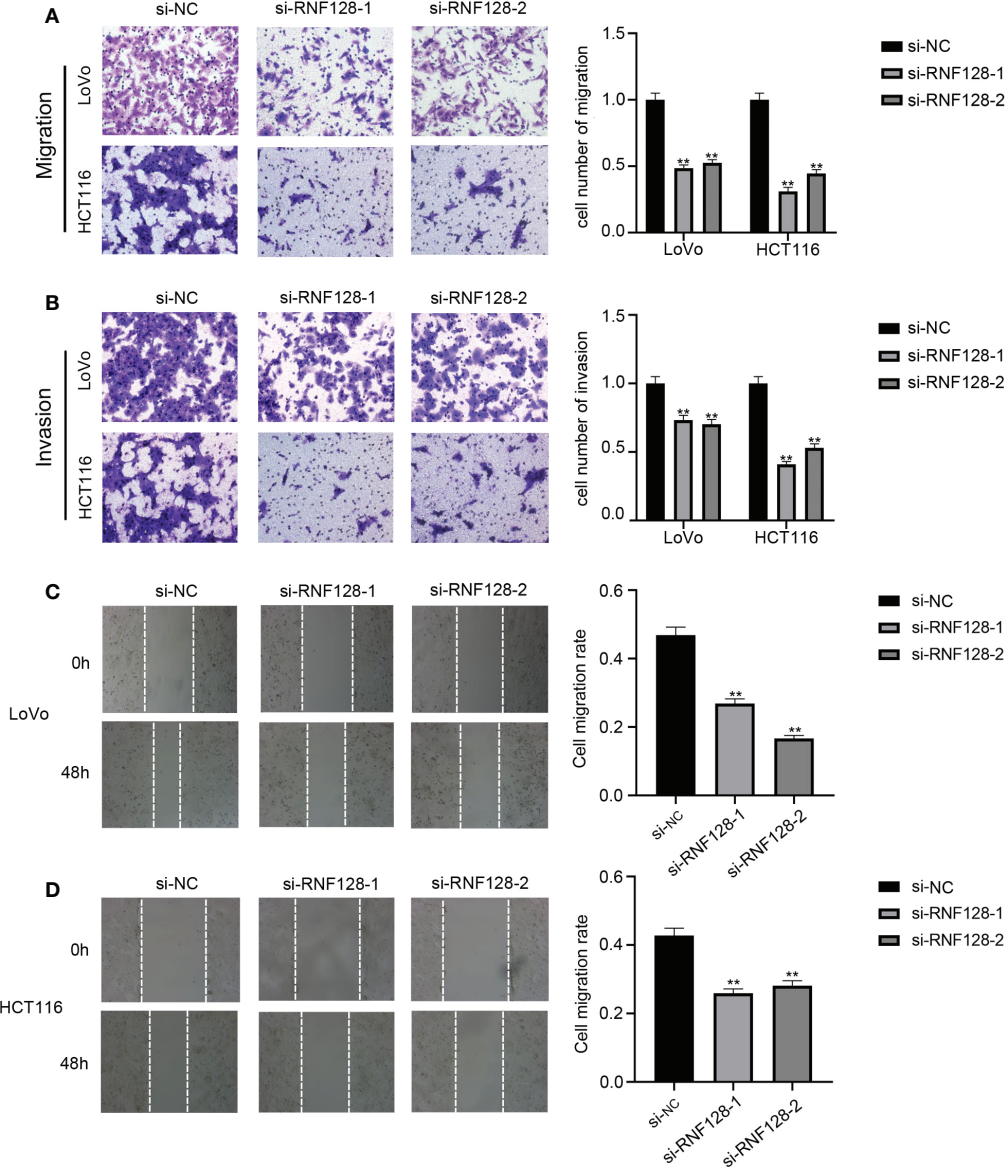
RNF128 promotes cell migration and invasion in colorectal cancer. **(A, C, D)** The migration abilities of LoVo and HCT116 cells were analyzed by transwell assays and wound healing assays with si-NC and si-RNF128 transfection. **(B)** The invasion abilities of LoVo and HCT116 cells transfected with si-NC and si-RNF128 were observed by transwell assays with Matrigel. **P<0.001.

To study whether RNF128 affects the migration and invasion of CRC cell lines, we conducted transwell and wound healing assays. Transwell assays demonstrated that the number of cells that traversed the membrane and Matrigel was significantly decreased with RNF128 knockdown (**Figures 3A, B**). In the wound healing assay, knockdown of RNF128 significantly reduced the rates of migration compared to those in the control groups in LoVo and HCT116 cells (**Figures 3C, D**). These results indicated that RNF128 may promote migration and invasion in CRC.

### RNF128 is involved in regulating the Hippo signaling pathway

**Figure 4:**
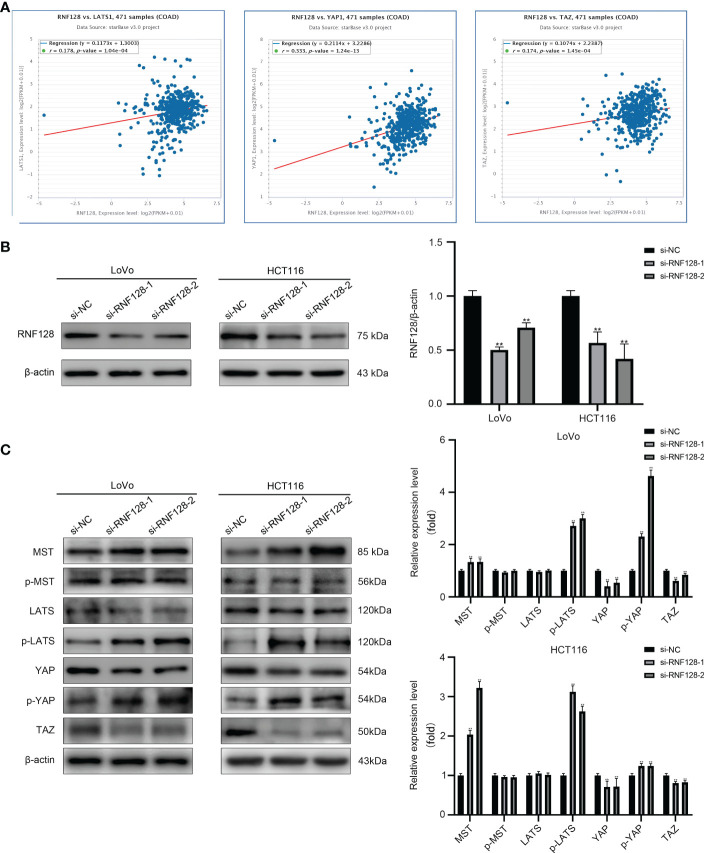
RNF128 inhibits the Hippo pathway in colorectal cancer. **(A)** RNF128 was correlated with Hippo pathway proteins (LATS, YAP, and TAZ) in CRC according to StarBase. **(B)** RNF128 was downregulated in LoVo and HCT116 cells transfected with si-NC and si-RNF128, as shown by western blot analysis. **P<0.001. **(C)** Western blot analysis was used to measure the protein expression of components of the Hippo pathway (MST, p-MST, LATS, p-LATS, YAP, p-YAP, and TAZ) in LoVo and HCT116 cells with RNF128 knockdown. **P<0.001.

The Hippo pathway plays a suppressive role in CRC progression. In this study, we found that RNF128 was strongly correlated with key proteins of the Hippo pathway (LATS, YAP, and TAZ) by StarBase prediction (**Figure 4A**). To further investigate whether RNF128 participates in regulating the Hippo pathway, we examined the expression of proteins in the Hippo signaling pathway by western blot analysis. The results showed that the expression of MST was upregulated with RNF128 knockdown and that p-LATS and p-YAP were hyperphosphorylated, whereas YAP and TAZ were downregulated in the HCT116 and LoVo cell lines; no significant changes were found in the expression levels of p-MST and LATS (**Figures 4B, C**). Therefore, we suggest that RNF128 may be involved in the development of CRC by regulating the Hippo pathway.

### RNF128 regulates MST protein stability and the expression of target genes of the Hippo pathway

**Figure 5:**
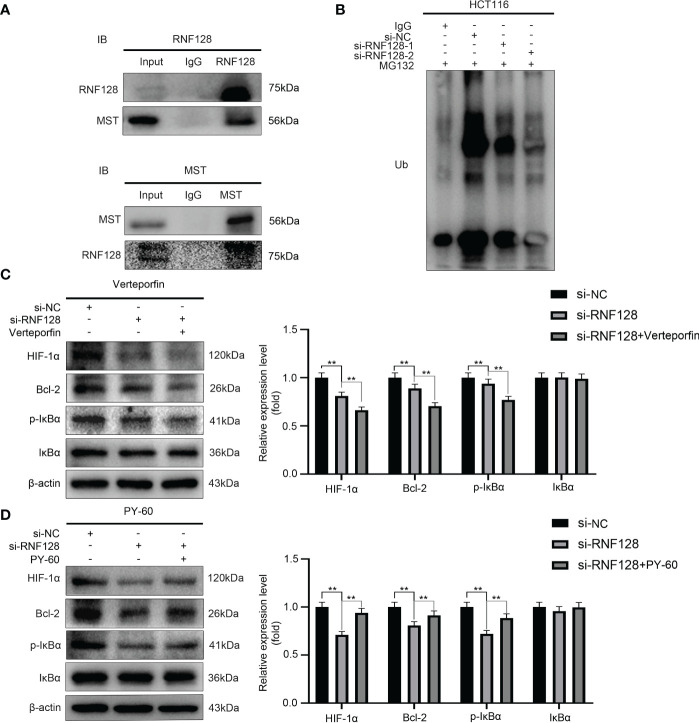
RNF128 ubiquitinates MST for degradation and regulates target genes of the Hippo signaling pathway. **(A)** Co-IP assays revealed that RNF128 interacts with MST. **(B)** An IP assay was used to examine the ubiquitination levels of MST. HCT116 cells were treated with MG132 (10 μM) for 6 h after transfection with si-NC and si-RNF128 for 48 h. **(C, D)** The expression levels of HIF-1α, Bcl-2, p-IκBα and IκBα were examined by western blotting. HCT116 cells were transfected with si-NC and si-RNF128 and treated with verteporfin (1 μM) for 16 h and PY-60 (10 μM) for 24 h. **P<0.001.

To further investigate how RNF128 regulated the Hippo signaling pathway, we used a co-IP assay to verify whether RNF128 bound to the proteins of the Hippo signaling pathway. The results showed that RNF128 interacted with MST (**Figure 5A**). Then, we examined how RNF128 regulates MST by an IP assay, the results showed that knockdown of RNF128 significantly decreased the ubiquitination level of MST compared to that in the si-NC group (**Figure 5B**). These results suggested that RNF128 regulates the Hippo signaling pathway by ubiquitinating MST to induce its degradation.

Studies have shown that the HIF-1α, Bcl-2 and the NF-κB signaling pathway components are targets of the Hippo pathway, and the activation and inactivation of the Hippo signaling pathway directly affect their expression levels (28–31). Therefore, we treated HCT116 cells with the YAP inhibitor verteporfin and the YAP agonist PY-60 after knocking down RNF128 and examined the expression levels of HIF-1α, Bcl-2, p-IκBα and IκBα. The Western blotting results showed that compared with those in the si-NC group, the expression levels of HIF-1α, Bcl-2, and p-IκBα in the si-RNF128 group were significantly decreased (**Figures 5C, D**). The expression levels of HIF1α, Bcl-2, and p-IκBα were significantly decreased in the si-RNF128+verteporfin group compared with the si-RNF128 group (**Figure 5C**), while the expression levels of HIF-1α, Bcl-2, and p-IκBα were significantly higher in the si-RNF128+PY-60 group than in the si-RNF128 group (**Figure 5D**), while IκBα expression levels did not show significant changes (**Figures 5C, D**). We suggest that RNF128 regulates downstream target genes through the Hippo signaling pathway.

### Knockdown of RNF128 inhibits the growth of colorectal cancer cells in nude mice

**Figure 6:**
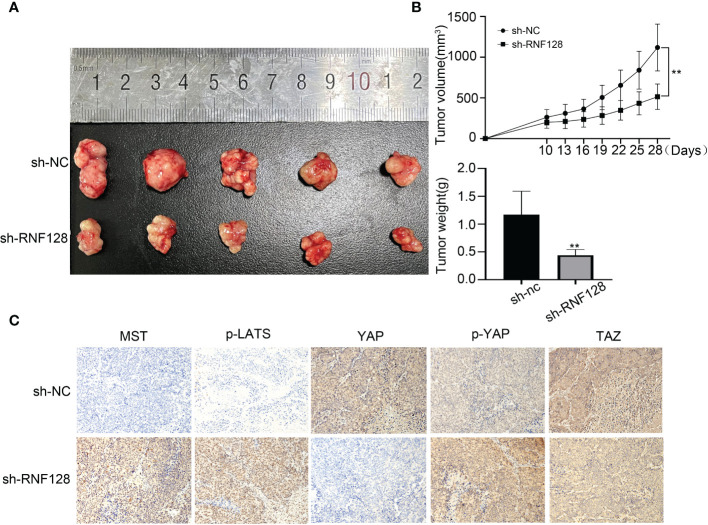
RNF128 promotes colorectal cancer progression in nude mice. **(A, B)** Tumors derived from nude mice injected with shNC and shRNF128 LoVo cells (n=5 per group). Tumor growth curves were generated, and tumor weight was measured. **P<0.001. **(C)** IHC analysis was used to assess the expression of Hippo pathway components in tumors.

To examine the effect of RNF128 on colorectal cancer cell growth *in vivo*, LoVo cells transfected with shRNF128 and shNC were inoculated into nude mice. Twenty-eight days after inoculation, tumors were removed from the nude mice. We found that RNF128 was closely related to tumor xenograft formation in nude mice, and these results confirmed that the tumor volume and weight in the shRNF128 group were lower than those in the shNC control group (**Figures 6A, B**). The IHC results were consistent with the *in vitro* western blotting results: the expression levels of MST, p-LATS and p-YAP were elevated in the shRNF128 group; conversely, the expression levels of YAP and TAZ were decreased (**Figure 6C**). Collectively, these results suggest that the Hippo pathway was activated after RNF128 was knocked down in CRC tissues.

## Discussion

In this study, we investigated the role of RNF128 in CRC progression. The results showed that RNF128 expression was significantly elevated in CRC and promoted the proliferation, migration and invasion of CRC cells by inhibiting the Hippo signaling pathway. Thus, our study suggests that RNF128 may be a novel target for the prevention and treatment of CRC.

RNF128 is a specific E3 ubiquitin ligase that is both a negative regulator of cytokine production and a unique mediator of CD4 T lymphocyte unresponsiveness (4, 6). Additionally, RNF128 is involved in the biological activities of lipopolysaccharide-induced hyperinflammation and organ damage, lipid accumulation and hepatic steatosis (7, 8, 32). Previous studies have demonstrated that RNF128 plays different roles in various cancers. In melanoma, low expression of RNF128 activates Wnt/β-catenin signaling to induce cellular EMT and stemness and protects CD44 and cortactin from degradation mediated by ubiquitination (12). Downregulation of RNF128 is also associated with a poor prognosis in patients with urothelial carcinoma of the upper tract and urinary bladder (13). In hepatocellular carcinoma and esophageal carcinoma, high expression of RNF128 promotes malignant behaviors in cancer cells through activation of the EGFR/ERK signaling pathway (10, 11).

A previous report demonstrated that RNF128 expression was significantly downregulated in CRC tissues and inhibited the tumor behavior of CRC cells by inhibiting the Wnt/β-catenin pathway (14). Interestingly, we found different expression statuses and mechanisms through analysis of cancer profiling databases and further experiments. Through GEPIA and TCGA analyses, we found that RNF128 expression was significantly higher in clinical CRC specimens than in adjacent normal tissues. Additionally, immunohistochemistry and western blot assays were carried out, and the results showed that RNF128 was dramatically highly expressed in CRC tissues and in HCT116 and LoVo cells. When compared with the control group, the proliferation, migration and invasion abilities of CRC cells were significantly decreased with RNF128 knockdown. In addition, in a mouse xenograft model, RNF128 knockdown suppressed the growth of CRC tumors. Based on experimental data and the cancer genome profiling data, it is reasonable to infer that RNF128 acts as a promoter, not a suppressor, during CRC progression and that RNF128 knockdown inhibits the proliferation, migration and invasion of CRC cells.

A large number of studies have shown that the Hippo signaling pathway functions in tumor immunity, energy homeostasis and cancer cell stemness to prevent cancer development (24, 33, 34). Due to these effects, the Hippo pathway is regarded as an important therapeutic target for breast, liver and colorectal cancers (23–26). In this study, we predicted the correlation between RNF128 and the Hippo pathway *via* StarBase, and the results showed that RNF128 was significantly correlated with the expression of LATS, YAP and TAZ. Then, we performed further experiments by western blotting and immunohistochemistry of nude mouse tumor tissues, which showed that RNF128 knockdown resulted in elevated expression of MST and increased phosphorylation of LATS and YAP, while the expression of YAP and TAZ was decreased. These results demonstrate that low expression of RNF128 contributes to activation of the Hippo signaling pathway and suppresses malignant tumor behavior in CRC.

As an E3 ubiquitin ligase, RNF128 can recognize and bind a variety of proteins to regulate cellular activity. Studies have demonstrated that RNF128 can interact with sirt1 and degrade it *via* ubiquitination to promote hepatocyte steatosis and that RNF128 also regulates innate antiviral immunity by promoting TBK1 ubiquitination (8, 35). In Th2 cells, RNF128 ubiquitinates and degrades STAT6 to participate in Th2 cell development (36). In the context of tumorigenesis, overexpression of RNF128 promotes p53 degradation and inhibits apoptosis (9). In our study, we found that RNF128 interacts with MST and regulates the Hippo signaling pathway through ubiquitination and degradation of MST. Knockdown of RNF128 in colorectal cancer cells followed by the application of the YAP inhibitor verteporfin and the YAP agonist PY-60 resulted in changes in the expression levels of target genes downstream of the Hippo signaling pathway, suggesting that RNF128 could regulate its downstream target genes through the Hippo signaling pathway, which consequently affects the malignant tumor behaviors of colorectal cancer cells.

In conclusion, our findings show that RNF128 expression is increased significantly in CRC tissues and that a high expression level of RNF128 promotes the proliferation, migration and invasion of CRC cells. Moreover, this study is the first to demonstrate that RNF128 can promote CRC development by inhibiting Hippo signaling pathway activation. These findings suggest that RNF128 may be a potential diagnostic and clinical therapeutic target for CRC patients.

## Data availability statement

The raw data supporting the conclusions of this article will be made available by the authors, without undue reservation.

## Ethics statement

The studies involving human participants were reviewed and approved by Ethical Committee of the Second Hospital of Dalian Medical University. The patients/participants provided their written informed consent to participate in this study. The animal study was reviewed and approved by Ethical Committee of the Second Hospital of Dalian Medical University.

## Author contributions

SN: experimental conception and design, write manuscript. YC: perform the experiment and data analysis. GW: help perform the analysis with constructive discussions. YL: help prepare the experimental material. YY: review and revision of the first draft, experimental supervision and guidance. ZZ: manage and coordinate experimental processes acquisition of the financial support for the project leading to this publication. All authors contributed to the article and approved the submitted version.
